# Supplementary material for: Clonal hematopoiesis related TET2 loss-of-function impedes IL1β-mediated epigenetic reprogramming in hematopoietic stem and progenitor cells
Source: Nat Commun. 2023 Dec 7;14:8102. doi: 10.1038/s41467-023-43697-y (PMC10703894; doi:10.1038/s41467-023-43697-y)
Supplement: Supplementary file 1 — Supplementary Information [file 41467_2023_43697_MOESM1_ESM.pdf]

Supplementary Information

Clonal hematopoiesis related TET2 loss-of-function impedes IL1β-mediated epigenetic reprogramming in hematopoietic stem and progenitor cells

McClatchy J.[1,2#], Strogantsev R.[3#], Wolfe E. [1,2], Lin HY.[1,2], Mohammadhosseini M.[1,2], Davis B.A. [1,2], Eden C.[1,2], Goldman D.[4,5], Fleming W.H.[4,5], Conley P[6], Wu G[6]. Cimmino L.[7], Mohammed H.[3*], Agarwal A.[1,2,3,4,8*§]

[1]Division of Oncological Sciences, Oregon Health & Science University, Portland, OR, USA
[2]Department of Cell, Developmental, and Cancer Biology, Oregon Health & Science University, Portland, OR, USA
[3]Cancer Early Detection Advanced Research Center, Knight Cancer Institute, Oregon Health & Science University, Portland, OR, USA
[4]Division of Hematology & Medical Oncology, Oregon Health & Science University, Portland, OR, USA
[5]Division of Pediatric Hematology and Oncology, Oregon Health & Science University, Portland, OR, USA
[6]Department of Medical Informatics and Clinical Epidemiology, Oregon Health & Science University, Portland, OR, USA
[7]University of Miami, Department of Biochemistry and Molecular Biology, Sylvester Comprehensive Cancer Center, Miami, USA
[8]Department of Molecular and Medical Genetics, Oregon Health & Science University, Portland, OR, USA

#These authors contributed equally
*These authors jointly supervised this work

[§]Correspondence:

Anupriya Agarwal, PhD
Mail Code: KR-HEM | 3181 S.W. Sam Jackson Park Road | Portland OR 97239
Ph: 503-494-7599 email: agarwala@ohsu.edu

INVENTORY FOR SUPPLEMENTAL INFORMATION

SUPPLEMENTAL FIGURES

SUPPLEMENTAL FIGURE 1s 4
Schematics for flow cytometric analysis of differentiated hematopoietic populations.

SUPPLEMENTAL FIGURE 2s 5
Chronic IL1β exposure enhances myelopoiesis at the expense of B cell frequency.

SUPPLEMENTAL FIGURE 3s 6
Within an inducible knockdown model chronic IL1β exposure increases the ratio of ly6chi to Ly6clo monocytes/macrophages.

SUPPLEMENTAL FIGURE 4s 8
Chronic IL1β exposure enhances myeloid expansion of Tet2-KO cells in non-competitive murine models.

SUPPLEMENTAL FIGURE 5s 10
IL1β stimulation promotes HSPC expansion in mice with Tet2 knockdown relative to WT in a competition repopulation experiment.

SUPPLEMENTAL FIGURE 6s 12
IL1β stimulation promotes myeloid bias of Tet2-KO CMPs and deletion of Tet2 within HSPCs is necessary for their expansion and myeloid bias.

SUPPLEMENTAL FIGURE 7s 13
IL1β-mediated expansion of Tet2-KO HSPCs is associated with reduced S/G2 frequency without significant differences in the frequency of apoptosis.

SUPPLEMENTAL FIGURE 8s 14
10x single cell RNA sequencing to identify cellular clusters from Tet2-KO and WT mice with and without IL1β stimulation in vivo.

SUPPLEMENTAL FIGURE 9s 16
Tet2-KO exaggerates frequency of GMPs over pseudotime.

SUPPLEMENTAL FIGURE 10s 18
Resistance to IL1β driven demethylation in Tet2-KO progenitors promotes differential hypermethylation associated with cellular fate.

SUPPLEMENTAL FIGURE 11s 20
Disruption of IL1R1 signaling suppresses myeloid bias in Tet2-KO mice.

SUPPLEMENTAL DATA

SUPPLEMENTAL DATA 1s (included separately as .xlsx file)
Cell number by sample.

SUPPLEMENTAL DATA 2s (included separately as .xlsx file)
Differentially expressed genes in Tet2-KO relative to WT HSCs.

SUPPLEMENTAL DATA 3s (included separately as .xlsx file)
Enrichr and STRING ontology analysis of Tet2-KO relative to WT HSCs.

SUPPLEMENTAL DATA 4s (included separately as .xlsx file)
Gene set enrichment analysis results for C2 molecular signature database in Tet2-KO relative to WT HSCs.

SUPPLEMENTAL DATA 5s (included separately as .xlsx file)
List of differentially methylated regions between Tet2-KO and WT LSK, CMP and GMP cells

SUPPLEMENTAL DATA 6s (included separately as .xlsx file)
List of GEO accession numbers for ChIP-seq factors.

SUPPLEMENTAL DATA 7s (included separately as .xlsx file)
HOMER motif analysis of differentially methylated regions between Tet2-KO and WT LSK, CMP and GMP cells.

Supplementary Figure 1

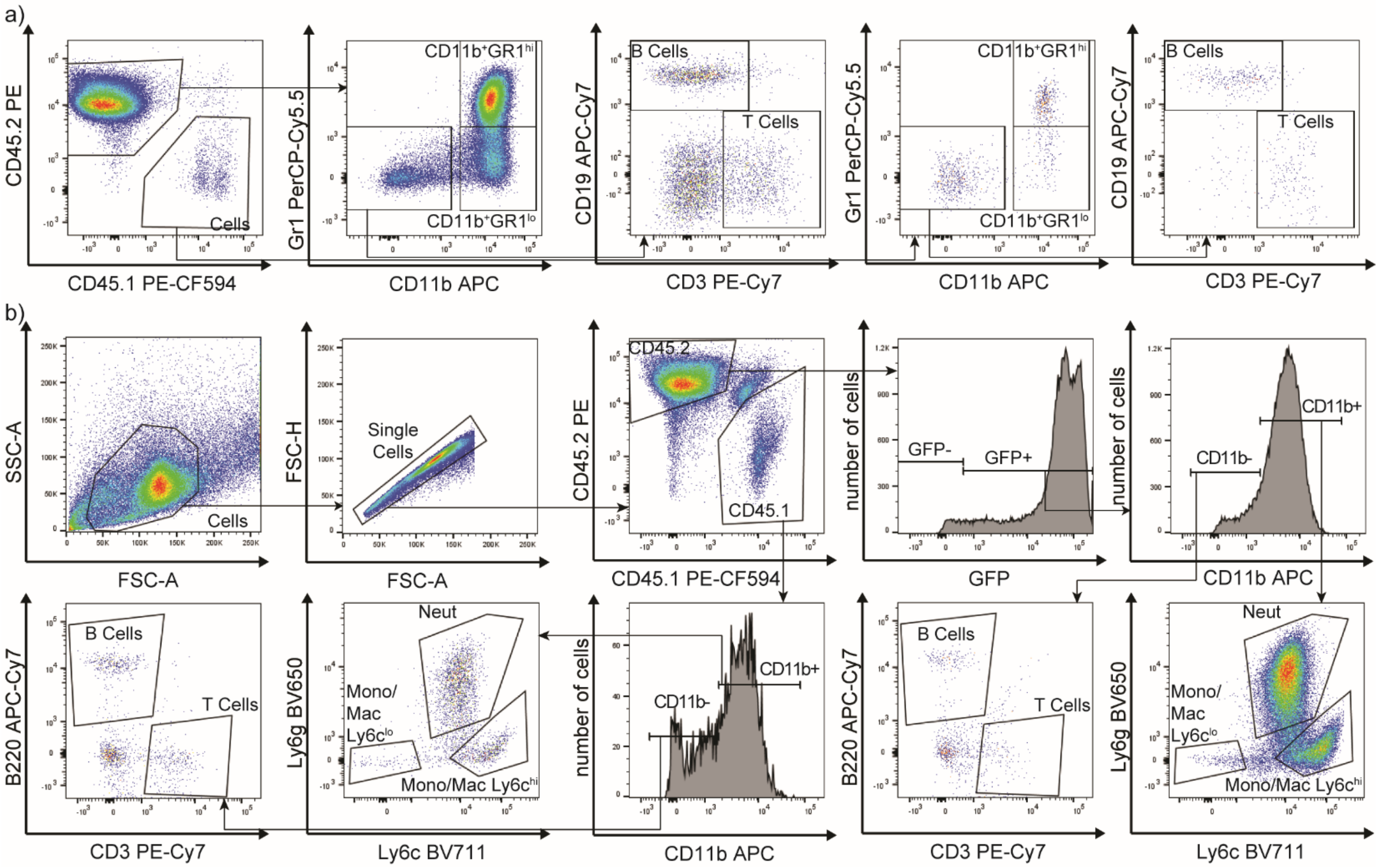

Supplementary Figure 1: Schematics for flow cytometric analysis of differentiated hematopoietic populations.

(a) Representative gating for differentiated subsets for flow cytometric analysis of the BM from chimeric mice, defining: $CD11b^{+}Gr1^{hi}$ ($CD11b^{+}Ly6g^{hi}Ly6c^{hi}$), $CD11b^{+}Gr1^{lo}$ ($CD11b^{+}Ly6g^{lo}Ly6c^{lo}$), B and T Cells (experimental design is shown in Fig. 1a). **(b)** Representative gating scheme for differentiated subsets for flow cytometric analysis of the BM from chimeric recipient mice, defining: neutrophils (Neut), $ly6c^{lo}$ monocytes/macrophages ($Ly6c^{lo}$ Mono/Mac), $ly6c^{hi}$ monocytes/macrophages ($Ly6c^{hi}$ Mono/Mac), B and T Cells (experimental design is shown in Fig. 1d).

Supplementary Figure 2

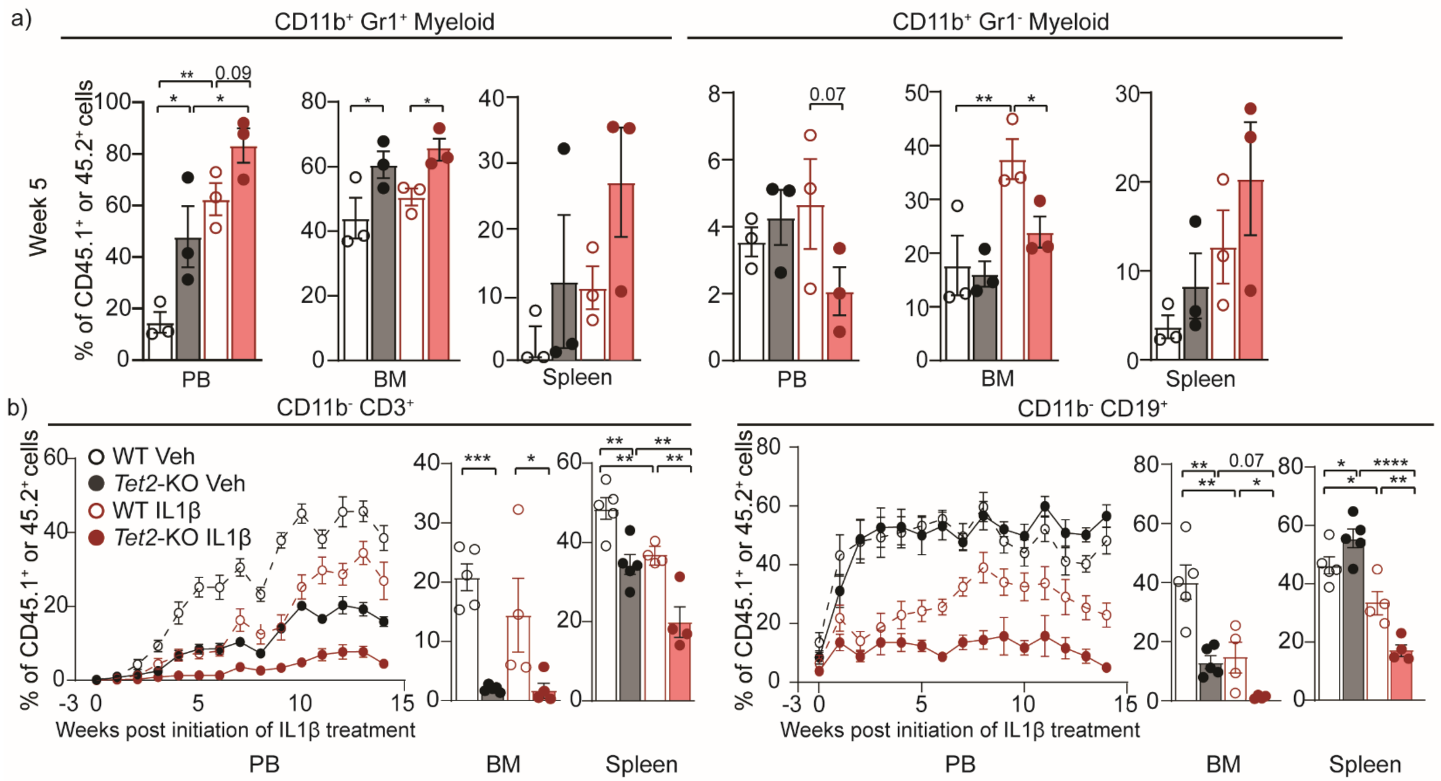

Supplementary Figure 2: Chronic IL1β exposure enhances myelopoiesis at the expense of B cell frequency.

(a-b) Lineage-depleted BM cells from WT CD45.1 and *Tet2*-KO CD45.2 mice were transplanted into lethally irradiated WT CD45.1/2 mice and after 3 weeks treated with IL1β (500 ng/mouse/day) or vehicle daily. **(a)** The frequency of myeloid subsets ($CD11b^{+}Gr1^{hi}$, $CD11b^{+}Gr1^{lo}$) at week 5 (n = 3 mice/group, week 15 is shown in main Fig. 1) and **(b)** T cells ($CD11b^{-}CD3^{+}$) and B cells ($CD11b^{-}CD19^{+}$) at 15 weeks in PB, BM, and spleen (n = 4-5 mice/group). Error bars represent mean ± SEM. Two-factor ANOVA was used for the FWER adjusted *p* values: $^{*}p < 0.05$, $^{**}p < 0.01$, $^{***}p < 0.001$, $^{****}p < 0.0001$.

Supplementary Figure 3

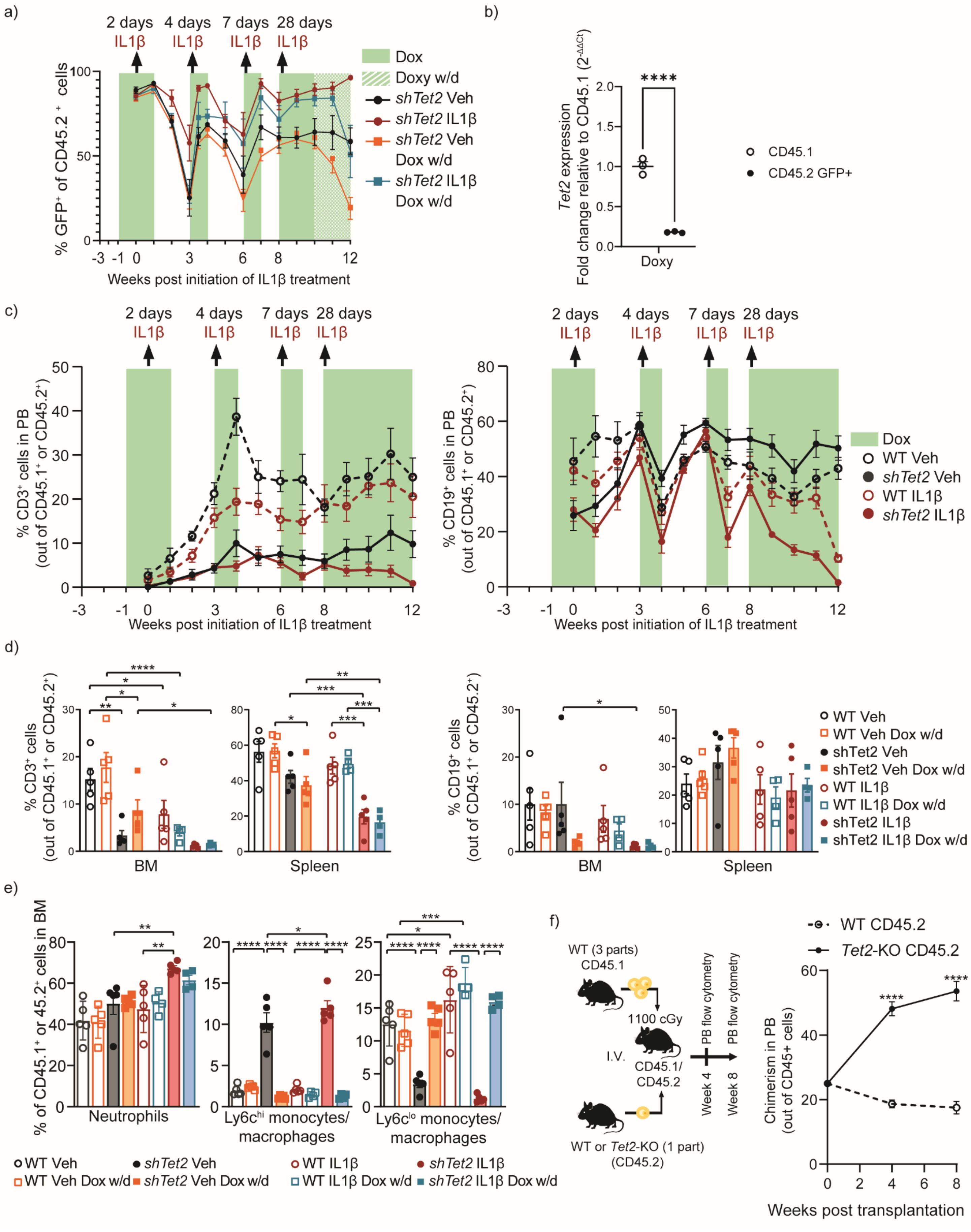

Supplementary Figure 3: Within an inducible knockdown model chronic IL1β exposure increases the ratio of ly6c^{hi} to Ly6c^{lo} monocytes/macrophages.

Lineage-depleted BM cells from wild-type (WT) CD45.1+ and Rosa-rtTA-driven inducible *Tet2* knockdown (*shTet2*) mice were transplanted into lethally irradiated WT CD45.1/2 mice, which were treated with doxycycline (dox) two weeks after transplantation and IL1β or vehicle three weeks after transplantation for increasing intervals up to 10 weeks and then with and without doxycycline (withdrawal, w/d) for an additional two weeks, analyzed by flow cytometry (n = 4-5 mice/group). Arrows indicate the time relative to the initial treatment (week: 0, 3, 6, 8) and duration of periods (days: 2, 4, 7, and 28) during which daily treatment with IL1β or vehicle occurred (n = 4-5 mice/group). The green color indicates the time and duration of doxycycline treatment, (described in Fig. 1d). **(a)** Percentage of CD45^{+} *shTet2* which are GFP^{+} in PB. **(b)** qPCR of *Tet2* expression from CD45.2^{+}GFP^{+} and CD45.1^{+} cells sorted from the BM of IL1β and doxycycline-treated mice (n = 3). **(c)** The percentage of WT CD45.1 or *shTet2* CD45.2 cells which are B (CD45^{+}CD11b^{-}B220^{+}) or T (CD45^{+}CD11b^{-}CD3^{+}) cells in the PB, **(d)** BM and Spleen **(e)** and the percentage of neutrophils (CD45^{+}CD11b^{+}Ly6c^{int}Ly6g^{+}), ly6c^{hi} monocytes/macrophages (CD45^{+}CD11b^{+}Ly6c^{+}Ly6g^{-}) and ly6c^{lo} monocytes/macrophages (CD45^{+}CD11b^{+}Ly6c^{-}Ly6g^{-}) in the BM (n = 4-5 mice/group). **(f)** Lineage-depleted BM from CD45.2 (WT or *Tet2*-KO) cells and WT CD45.1 cells were transplanted into lethally irradiated WT CD45.1/2 recipients in a 1 to 3 ratio and analyzed 4 and 8 weeks post-transplantation by flow cytometry (n = 8 CD45.1 and n = 28 CD45.2 mice/group). Error bars represent mean ± SEM. For panel b, d, and e, two-factor ANOVA was used to determine the FWER-adjusted *p* values. For panel f a student's two-tailed t-Test was used to determine significance. For family-wise error rate (FWER) adjusted and regular *p* values: *p < 0.05, **p < 0.01, ***p < 0.001, ****p < 0.0001.

Supplementary Figure 4

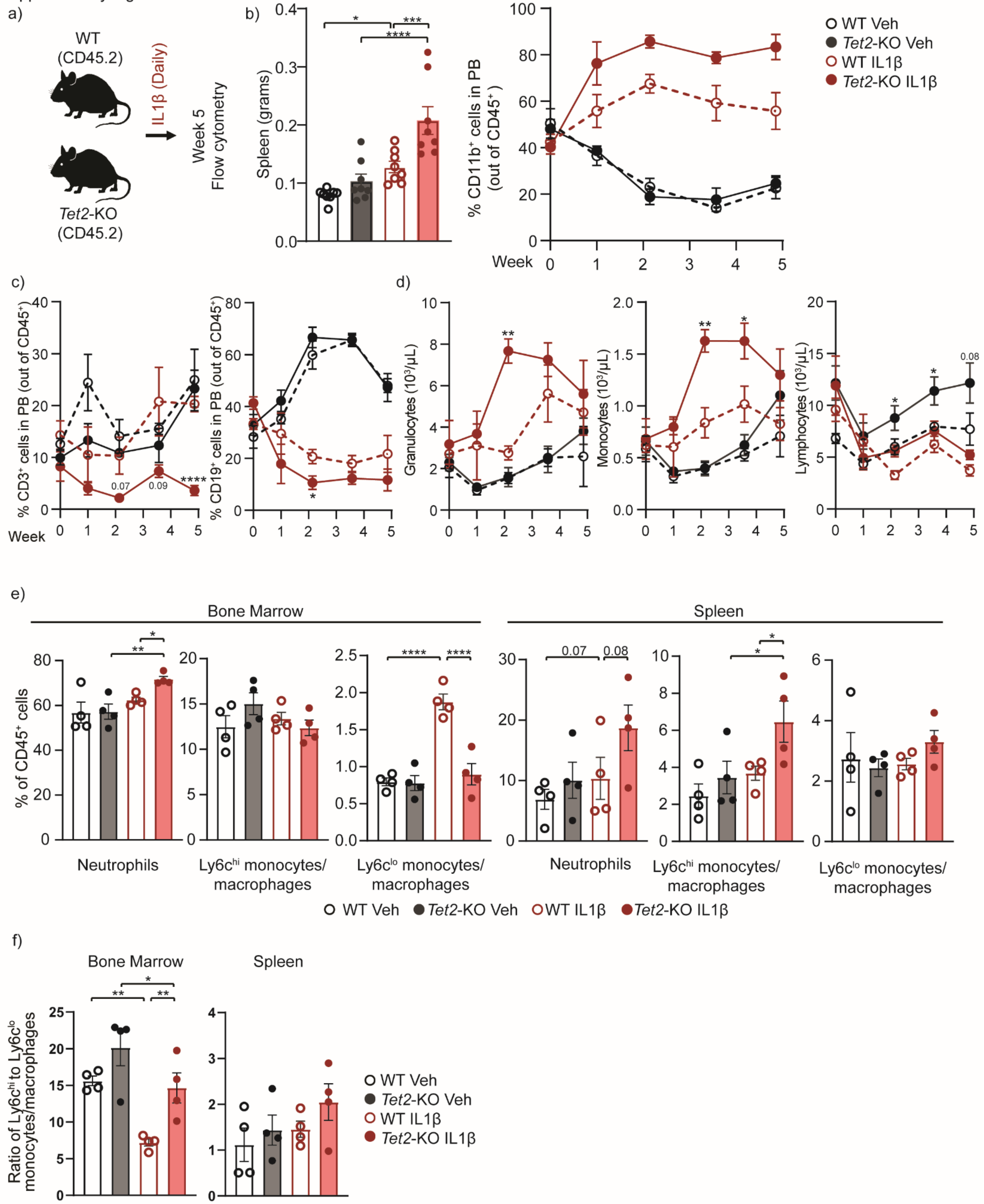

Supplementary Figure 4: Chronic IL1β exposure enhances myeloid expansion of *Tet2*-KO cells in non-competitive murine models.

Tet2$^{fl/fl}$ (WT) and *Vav-Cre Tet2$^{fl/fl}$* (*Tet2*-KO) mice were treated with IL1β (500 ng/mouse/day) or vehicle (n = 4 mice/group) for five weeks and peripheral blood (PB), bone marrow (BM) and spleen samples were analyzed by flow cytometry. **(a)** Experimental design, **(b)** spleen weight in grams (left) and the percentage of the PB $CD45^{+}$ cells which are myeloid ($CD45^{+}CD11b^{+}$, right), **(c)** T Cells or B Cells. **(d)** Differential blood cell counts representing granulocytes, monocytes, and lymphocytes in the PB. **(e)** Neutrophils, $ly6c^{hi}$ monocytes/macrophages, and $ly6c^{lo}$ monocytes/macrophages and **(f)** ratio of $Ly6c^{hi}$ to $ly6c^{lo}$ monocytes/macrophages in BM and spleen cells at week five by flow cytometry. Error bars represent mean ± SEM. For panel b (left), d and e two-factor ANOVA, was used to determine FWER adjusted p values. For panel b (right) and c a student's two-tailed t-Test was used to determine significance. For FWER adjusted and regular *p* values: *p < 0.05, **p < 0.01, ***p < 0.001, ****p < 0.0001.

Supplementary Figure 5

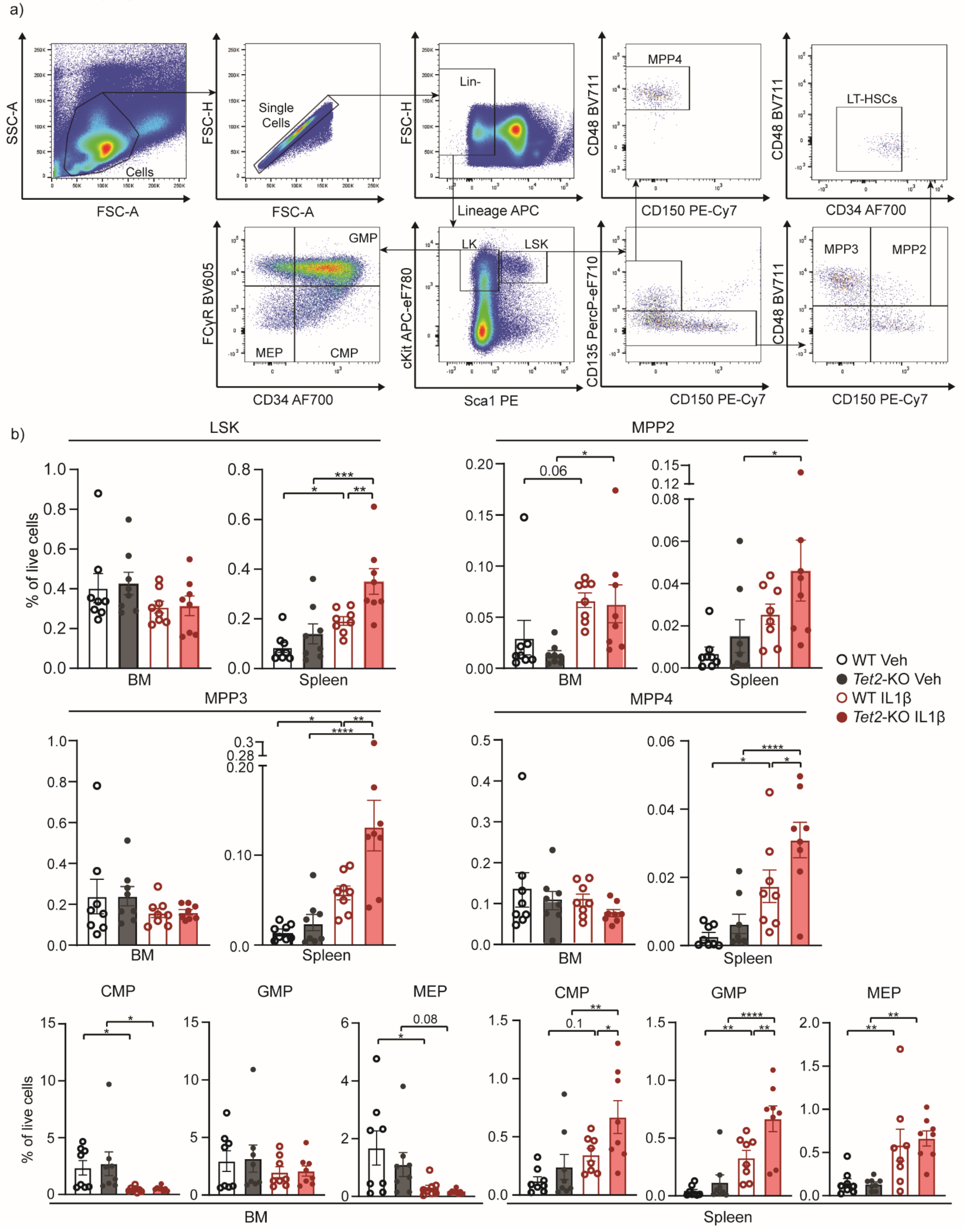

Supplementary Figure 5: IL1β stimulation promotes HSPC expansion in mice with *Tet2* knockdown relative to WT in a competition repopulation experiment.

(a) Representative gating scheme for HSPCs for flow cytometric analysis of the BM from a WT mouse. **(b)** WT and *Tet2*-KO mice were treated with IL1β or vehicle for five weeks (Supplementary Fig. 4a) and analyzed by flow cytometry for the percentage of live $CD45^{+}$ cells which are LSKs, MPP2, MPP3, MPP4, CMP, GMP and MEP in the spleen or BM (4 mice/group from two separate experiments, total n = 8 mice/group). Error bars represent mean ± SEM. Two factor ANOVA was used to determine the FWER adjusted p values: *$p < 0.05$, **$p < 0.01$, ***$p < 0.001$, ****$p < 0.0001$.

Supplementary Figure 6

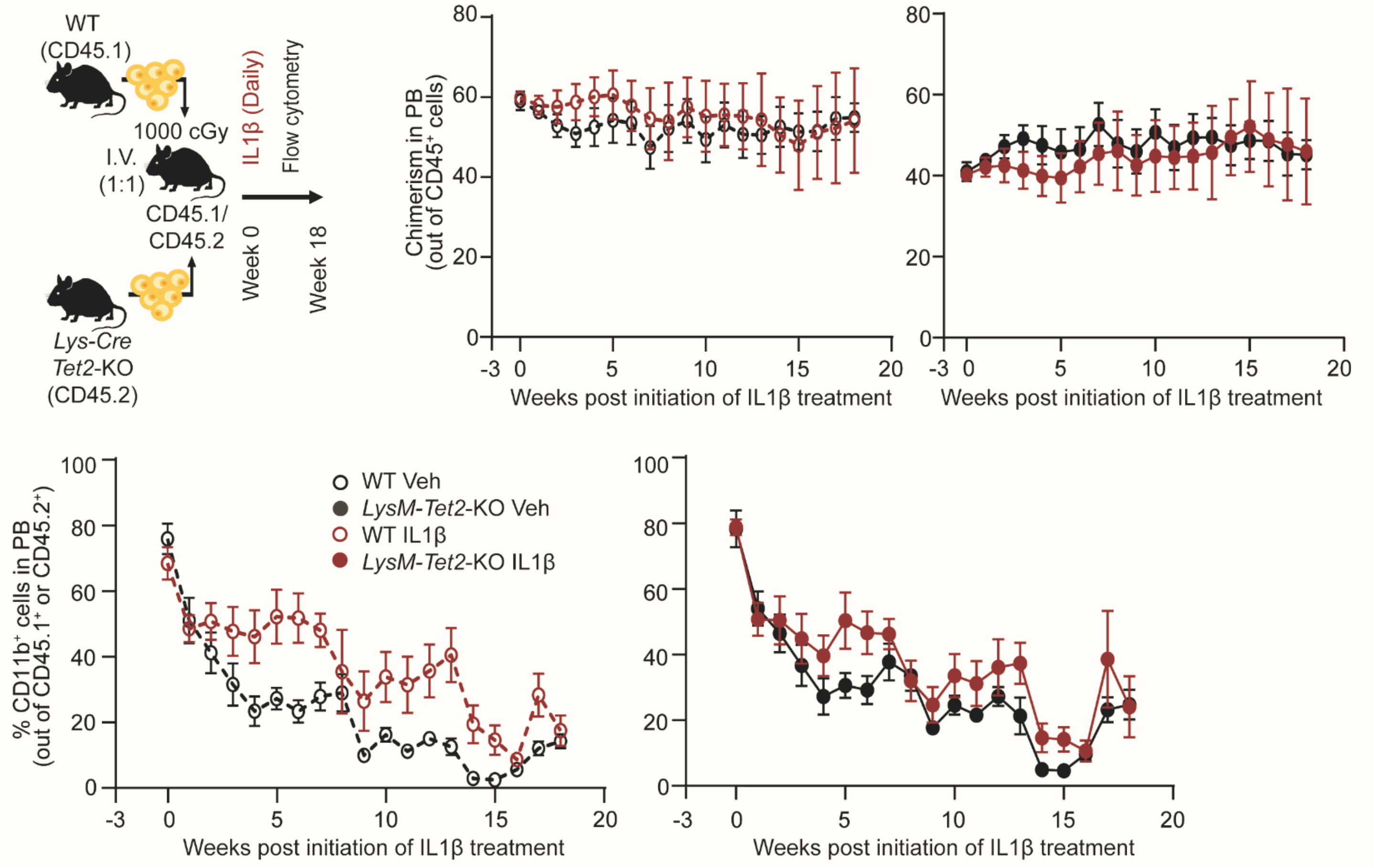

Supplementary Figure 6: IL1β stimulation promotes myeloid bias of *Tet2*-KO CMPs and deletion of *Tet2* within HSPCs is necessary for their expansion and myeloid bias.

Lineage-depleted BM cells were harvested from WT CD45.1 and *LysM-Cre Tet2*$^{-/-}$ CD45.2 (*LysM-Cre Tet2*-KO) mice into lethally irradiated WT CD45.1^{+}CD45.2^{+} mice which after 3 weeks were treated with IL1β or vehicle. The percentage of WT CD45.1 and *LysM-Cre Tet2*-KO CD45.2 cells out of all CD45^{+} cells (chimerism) and the percentage WT CD45.1 and *Tet2*-KO CD45.2 cells which are myeloid in the PB. Error bars represent mean ± SEM. Student's two-tailed t-Test was used to determine significance.

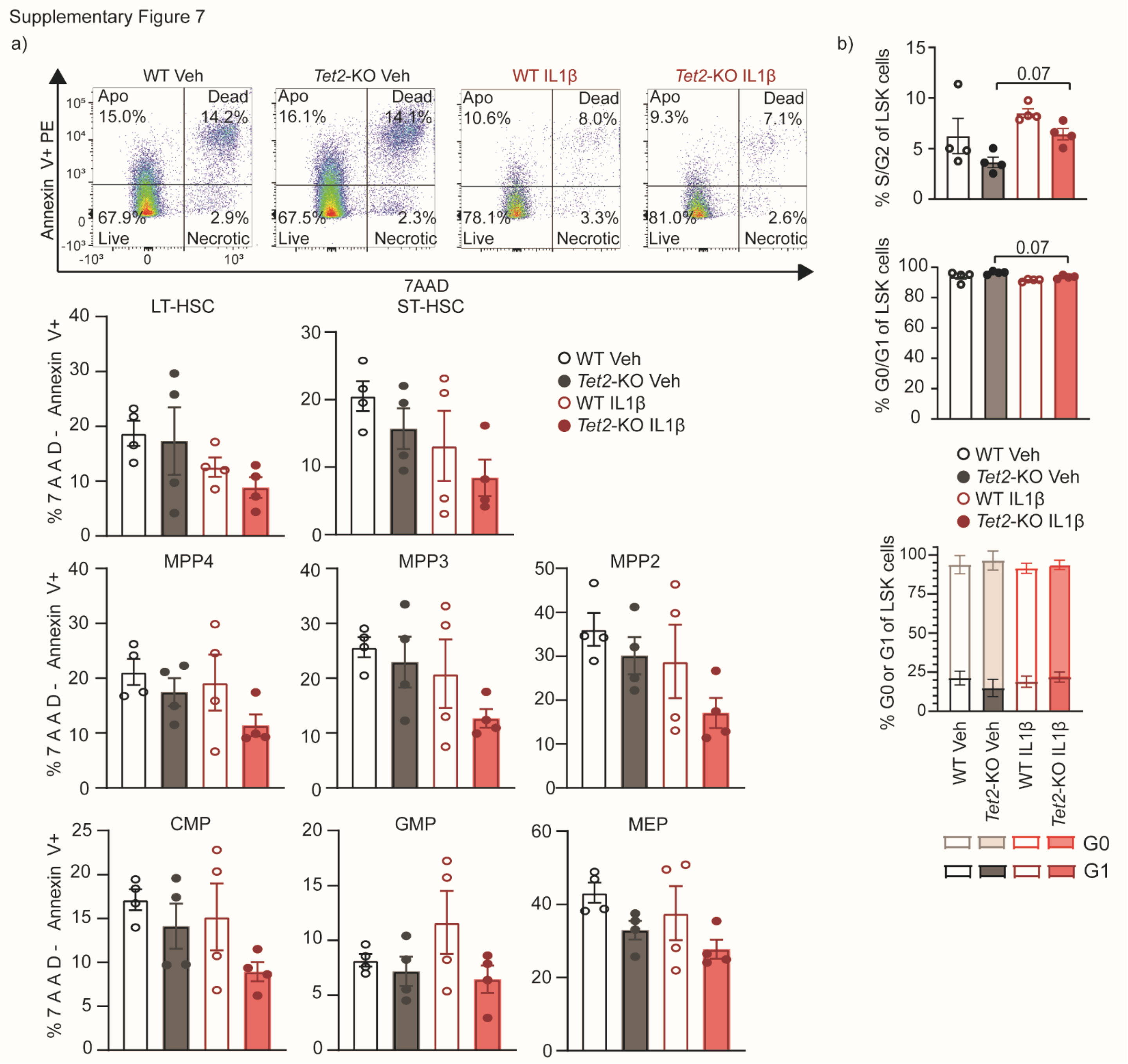

Supplementary Figure 7: IL1β-mediated expansion of *Tet2*-KO HSPCs is associated with reduced S/G₂ frequency without significant differences in the frequency of apoptosis.

(a-b) WT and *Tet2*-KO mice were treated with IL1β for 5 weeks (as described in Supplementary Fig. 4a; n = 4 mice/group). The percentage of apoptotic ($7AAD^{-}Ann\ V^{+}$) cells for HSPC subsets was quantified by flow cytometry. **(b)** Flow cytometry analysis of LSK cells isolated from the BM of *Tet2*-KO and WT mice treated with and without IL1β for 5 weeks, with cell cycle proportions using KI-67 and DAPI (right, n = 4 mice/group). Error bars represent mean ± SEM. Two-factor ANOVA was used to determine the FWER adjusted *p* values.

Supplementary Figure 8

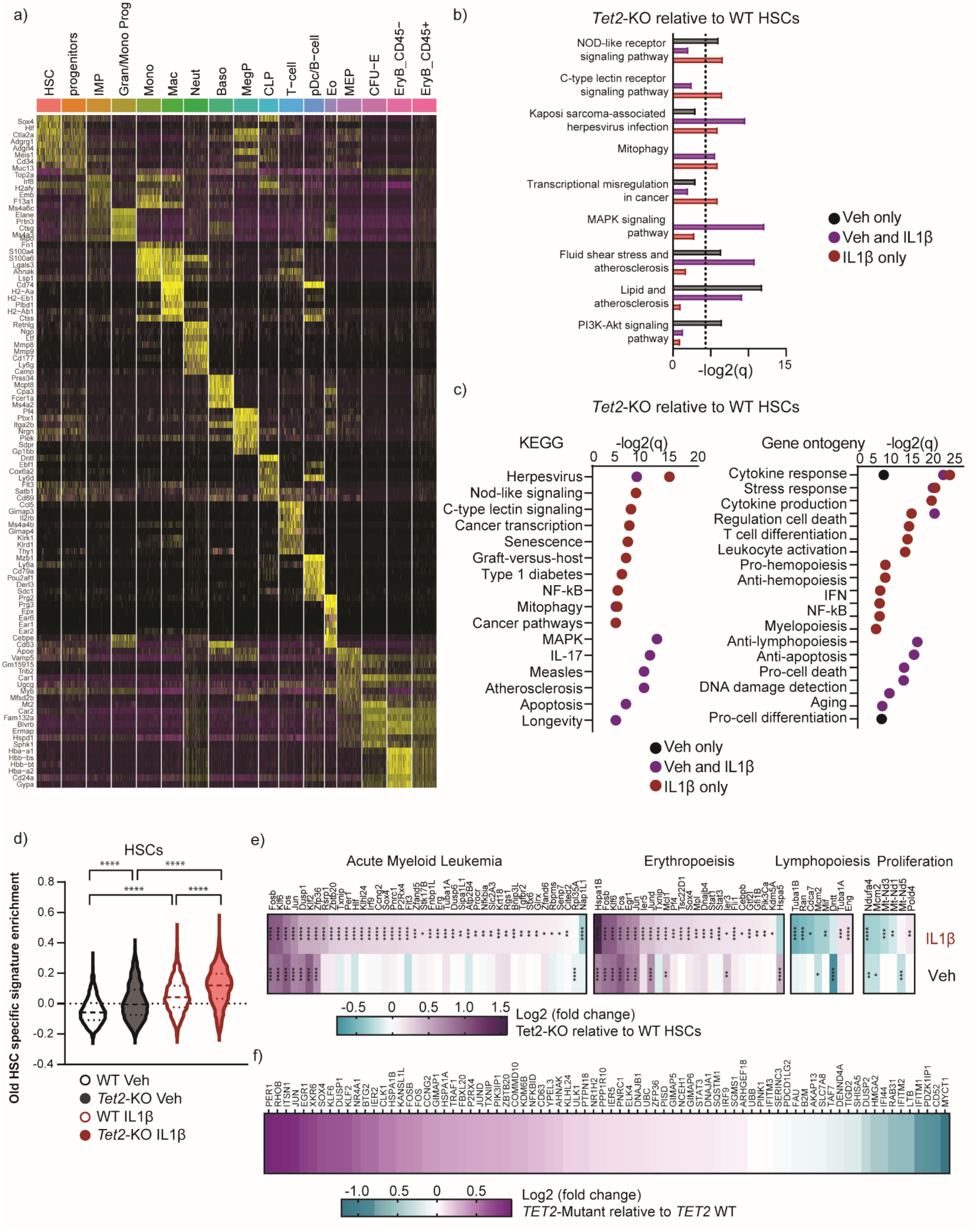

Supplementary Figure 8: 10x single cell RNA sequencing to identify cellular clusters from *Tet2*-KO and WT mice with and without IL1β stimulation *in vivo*.

(a) Heatmap showing gene expression of cluster-defining genes for individual cells from each cluster. (**b)** Enrichr ontology (KEGG 2021 human) of DEGs upregulated in *Tet2*-KO relative to WT HSCs in vehicle alone (black), IL1β alone (red) or in both (purple) with q values calculated by Enrichr. **(c)** STRING KEGG response to cytokine from biological process gene ontology (GO:0034097) and MAPK signaling pathway from KEGG Pathways (mmu04010) of DEGs upregulated in *Tet2*-KO relative to WT only under vehicle alone (black), IL1β alone (red), or both conditions (purple) with q values calculated by STRING. **(d)** Enrichment of transcriptional signature specific to old HSCs (Kirschner et al., Cell Rep, 2017) within HSCs. **(e)** Heatmap of upregulated DEGs in *Tet2*-KO relative to WT HSCs with and without IL1β stimulation. The genes shown are the lead genes for enriched pathways by GSEA analysis, with genes identified in multiple pathways shown multiple times. *q* values were determined by DESeq2. **(f)** Differential gene expression in *TET2*-mutant relative to *TET2*-WT de novo primary AML for the top 100 human orthologous genes that are upregulated in *Tet2*-KO relative to WT murine HSCs stimulated with IL1β. For q values: *q < 0.05, **q < 0.01, ***q < 0.001, ****q < 0.0001.

Supplementary Figure 9

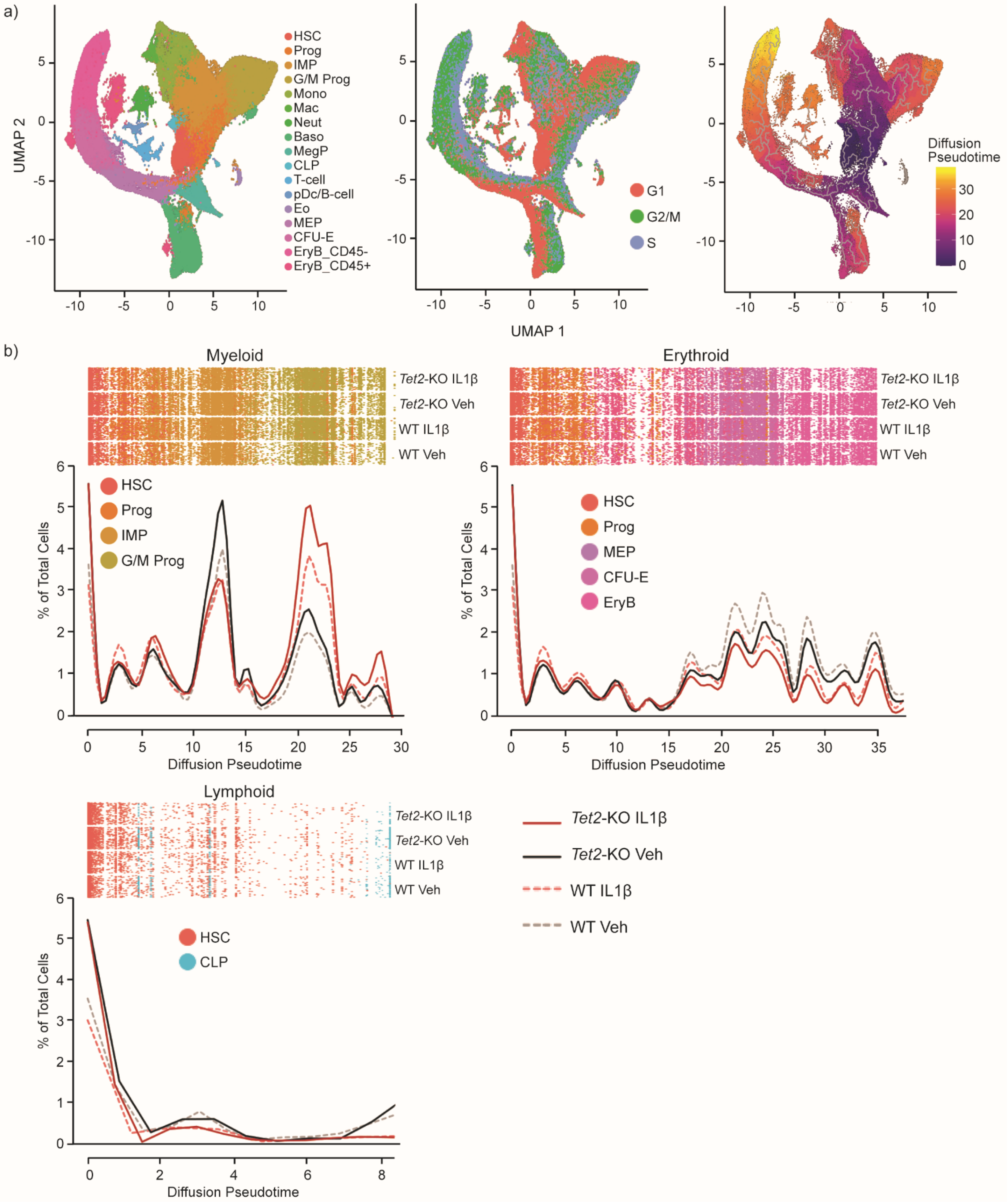

Supplementary Figure 9: *Tet2*-KO exaggerates frequency of GMPs over pseudotime.

Cells analyzed by 10X single cell RNA (scRNA) sequencing (described in Figure 3a) were assigned pseudotime values using Monocle3. **(a)** UMAP from cell cycle G0/1, S, G2 regressed populations, showing clusters labelled as in Figure 3b (left), Cell Cycle (middle), and Monocle3 derived pseudotime values (right). **(b)** Pseudotime as HSCs differentiate over myeloid (HSC, Prog, IMP, GMP), erythroid (HSC, Prog, MEP, CFU-E, EryB) and lymphoid (HSC, CLP) lineage trajectories.

Supplementary Figure 10

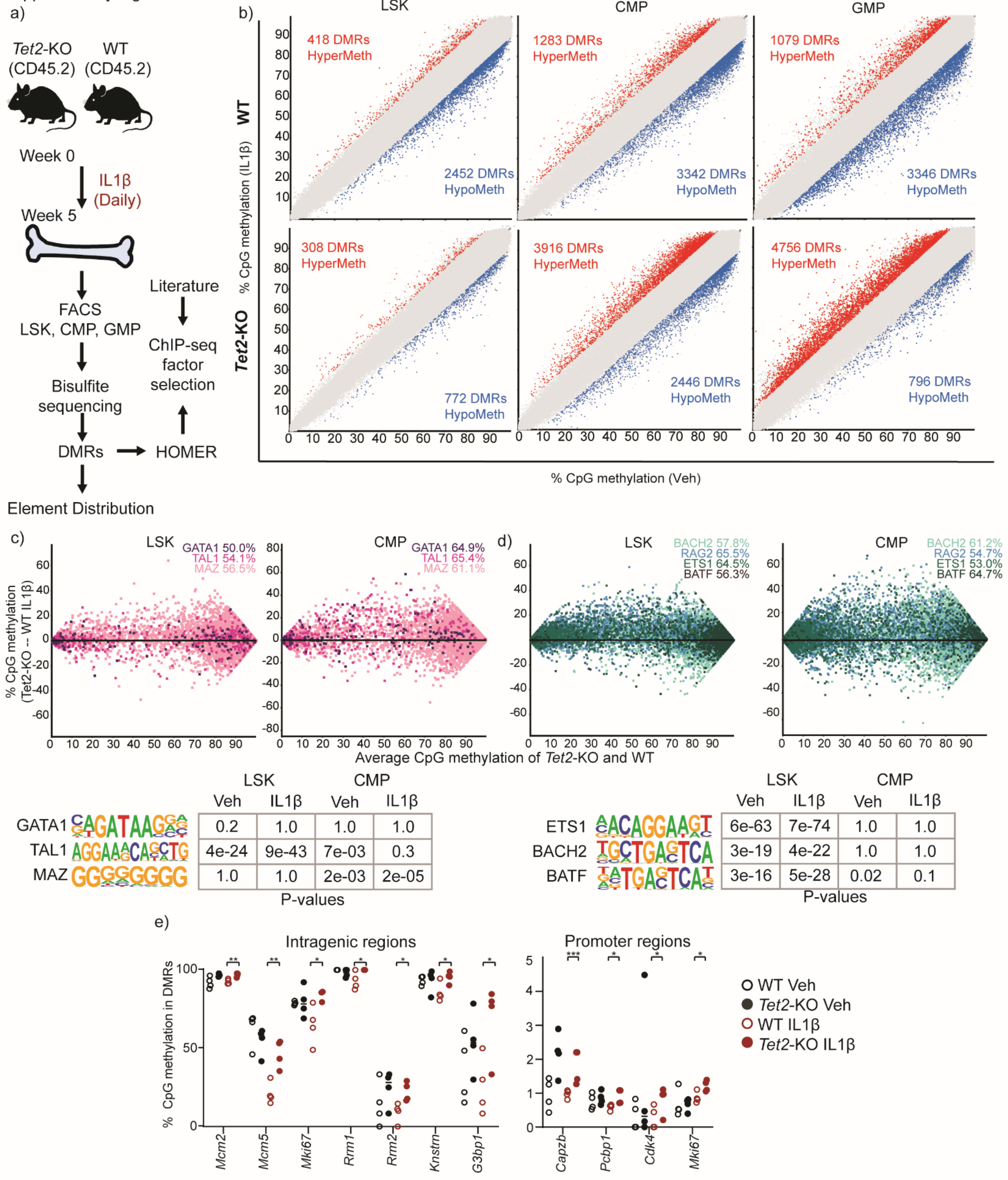

	LSK Veh	LSK IL1β	CMP Veh	CMP IL1β
GATA1	0.2	1.0	1.0	1.0
TAL1	4e-24	9e-43	7e-03	0.3
MAZ	1.0	1.0	2e-03	2e-05

P-values

	LSK Veh	LSK IL1β	CMP Veh	CMP IL1β
ETS1	6e-63	7e-74	1.0	1.0
BACH2	3e-19	4e-22	1.0	1.0
BATF	3e-16	5e-28	0.02	0.1

P-values

Supplementary Figure 10: Resistance to IL1β driven demethylation in *Tet2*-KO progenitors promotes differential hypermethylation associated with cellular fate.

WT and *Tet2*-KO mice were treated for 5 weeks with or without IL1β, purified BM-derived LSK, CMP, and GMP cells were analyzed by whole genome bisulfite sequencing (n = 4 mice/group). **(a)** Experimental design, **(b)** differentially methylated regions (DMRs) from 50 CpGs tiling of the whole genome (>10% methylation difference, q < 0.05, as determined by SeqMonk) between vehicle or IL1β treatment in WT or *Tet2*-KO cells. **(c)** Top, MA plots showing methylation difference for LSK and CMP cells derived from *Tet2*-KO and WT mice treated with IL1β using literature-derived ChIP-seq binding sites of erythroid/megakaryocytic lineage specifying transcription factors (TFs). The percentage of sites with higher methylation in *Tet2*-KO than WT are shown in the top right corner of each graph. Bottom, the significance of motif enrichment using HOMER analysis tool within DMRs for TFs. With q values calculated by Homer. **(d)** As in Supplementary Fig. 10c, but showing lymphoid lineage specifying TFs. **(e)** Selected examples of genes showing methylation differences within promotor (top) or intragenic (bottom) regions. Student's two-tailed t-test was used to determine significance except where otherwise specified in the figure legend: *$p < 0.05$, **$p < 0.01$, ***$p < 0.001$.

Supplementary Figure 11

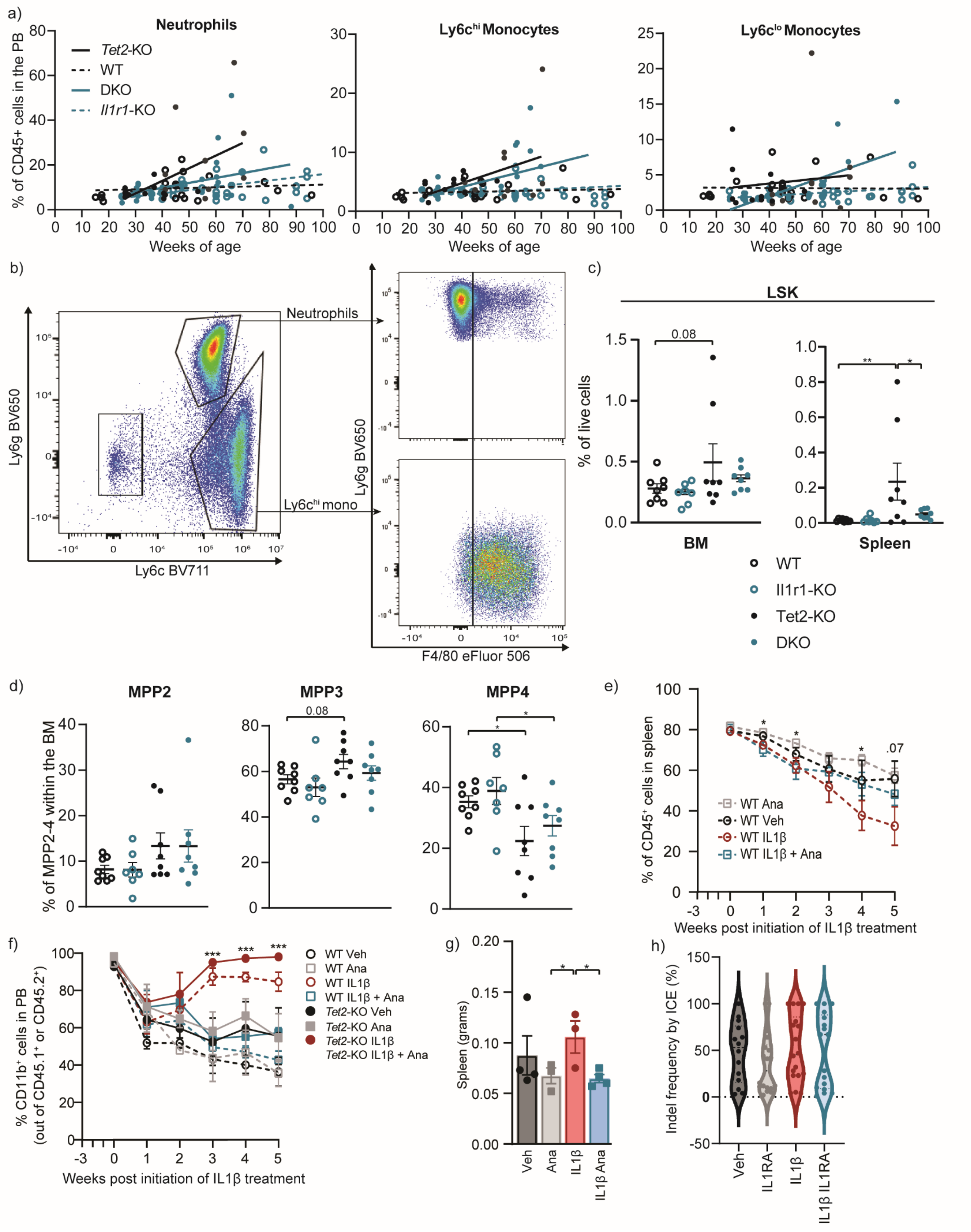

Supplementary Figure 11: Disruption of IL1R1 signaling suppresses myeloid bias in *Tet2*-KO mice.

(a-d) *Vav-cre Tet2$^{fl/fl}$* (*Tet2*-KO), *Il1r1$^{-/-}$* (*Il1r1*-KO), and *Vav-cre Tet2$^{fl/fl}$Il1r1$^{-/-}$* (double knockout; DKO) mice over 60 weeks of age. **(a)** Frequency of neutrophils, Ly6c^{hi} monocytes, and Ly6c^{lo} monocytes in the PB. **(b)** Representative gating within BM of the percentage of Ly6c^{hi} monocytes/macrophages which are F4/80^{+}. **(c)** The frequency out of live cells of LSK cells within the BM and spleen. **(d)** Frequency out of live cells of MPP2, MPP3, and MPP4 out of MPP2-4 within the BM. **(e-g)** Lineage-depleted BM cells derived from WT CD45.1 and *Tet2*-KO CD45.2 mice were transplanted into lethally irradiated WT CD45.1/2 mice. 3 weeks later, mice were treated with IL1β (500 ng/mouse/day) or vehicle as well as with or without an IL1R1 antagonist (anakinra, 100mg/kg) (n = 3-4 mice/group). **(e)** Percentage of WT CD45.1 donor cells out of all CD45^{+} cells (chimerism) in PB. **(f)** Percentage of WT CD45.1 and *Tet2*-KO CD45.2 donor cells which are myeloid in the PB. **(g)** Spleen weight in grams. **(h)** Indel frequency of individually picked colonies for vehicle, IL1RA, IL1β, and IL1β with IL1RA is determined by Sanger sequencing for infer CRISPR edits (ICE) analysis. Error bars represent mean ± SEM. For panels c and d, two-factor ANOVA was used to determine FWER-adjusted p values. For panels g and h, one factor was used to determine the FWER-adjusted *p* values. For panels e and f, a student's two-tailed t-Test was used to determine significance. For FWER adjusted and regular *p* values: *p < 0.05, **p < 0.01, ***p < 0.001, ****p < 0.0001.

Supplementary Data 1: Cell number by sample. WT and *Tet2*-KO mice were treated for 5 weeks with and without IL1β (n = 4 mice/group). BM cells were harvested and lineage-negative cells were enriched by magnetic selection. Cells were analyzed by 10X single cell RNA (scRNA) sequencing as described in figure 3a. The data shows cell number metadata such as cell number per mouse per cell type.

Supplementary Data 2: Differentially expressed genes in *Tet2*-KO relative to WT HSCs. WT and *Tet2*-KO mice were treated for 5 weeks with and without IL1β (n = 4 mice/group). BM cells were harvested and lineage-negative cells were enriched by magnetic selection. Cells were analyzed by 10X single cell RNA (scRNA) sequencing as described in figure 3a. The data shows differential gene expression with a q value of 0.05 cut off between *Tet2*-KO and WT HSCs as determined using DESeq2.

Supplementary Data 3: Enrichr and STRING ontology analysis in *Tet2*-KO relative to WT HSCs. WT and *Tet2*-KO mice were treated for 5 weeks with and without IL1β (n = 4 mice/group). BM cells were harvested and lineage cells were depleted by magnetic selection. Cells were analyzed by 10X single cell RNA (scRNA) sequencing as described in figure 3a. Enrichr (KEGG 2021 Human), STRING KEGG Pathways (mmu04010), or STRING biological process gene ontology (GO:0034097) identified pathways from genes upregulated in *Tet2*-KO relative to WT HSCs treated with or without IL1β, or in both conditions. q values were determined through Enrichr and STRING.

Supplementary Data 4: Gene set enrichment analysis results for C2 molecular signature database in *Tet2*-KO relative to WT HSCs. WT and *Tet2*-KO mice were treated for 5 weeks with and without IL1β (n = 4 mice/group). BM cells were harvested and lineage cells were depleted by magnetic selection. Cells were analyzed by 10X single cell RNA (scRNA) sequencing as described in figure 3a. Gene set enrichment analysis (C2) of differential expressed genes between HSCs in *Tet2*-KO and WT with and without IL1β administration. q values were determined by GSEA v4.2.1 using an empirical phenotype-based permutation test procedure.

Supplementary Data 5: List of differentially methylated regions between *Tet2*-KO and WT LSK, CMP and GMP cells. WT and *Tet2*-KO mice were treated for 5 weeks with and without IL1β (n = 4 mice/group). BM cells were harvested and LSK, CMP and GMP cells were flow sorted and bisulfite sequenced as described in Supplementary Figure 7a. Data shows differentially methylated regions in *Tet2*-KO relative to WT LSK, CMP, and GMP cells in *Tet2*-KO relative to WT with and without IL1β administration and in the vehicle, relative IL1β treated mice. >10% methylation difference, q < 0.05, statistical significance determined by SeqMonk.

Supplementary Data 6: List of GEO accession numbers for ChIP-seq factors. GEO accession numbers for ChIP-seq databases whose called peaks were acquired from cistrome.

Supplementary Data 7: HOMER motif analysis of differentially methylated regions between *Tet2*-KO and WT LSK cells. Differentially methylated regions for fluorescently activated cell sorted LSK, CMP, and GMP cells described in Supplementary Data 4 underwent HOMER motif analysis for *Tet2*-KO relative to WT mice treated with vehicle or IL1β. Statistical significance of motif enrichment was determined by hypergeometric distribution using the HOMER analysis tool.
