# Supplementary material for: Clonal hematopoiesis related TET2 loss-of-function impedes IL1β-mediated epigenetic reprogramming in hematopoietic stem and progenitor cells
Source: Nat Commun. 2023 Dec 7;14:8102. doi: 10.1038/s41467-023-43697-y (PMC10703894; doi:10.1038/s41467-023-43697-y)
Supplement: Supplementary file 3 — Description of Additional Supplementary Files [file 41467_2023_43697_MOESM3_ESM.pdf]

Description of Additional Supplementary Files

File Name: Supplementary Data 1
Description: **Cell number by sample.** WT and *Tet2*-KO mice were treated for 5 weeks with and without IL1β (n = 4 mice/group). BM cells were harvested and lineage-negative cells were enriched by magnetic selection. Cells were analyzed by 10X single cell RNA (scRNA) sequencing as described in figure 3a. The data shows cell number metadata such as cell number per mouse per cell type.

File Name: Supplementary Data 2
Description: **Differentially expressed genes in Tet2-KO relative to WT HSCs.** WT and *Tet2*-KO mice were treated for 5 weeks with and without IL1β (n = 4 mice/group). BM cells were harvested and lineage-negative cells were enriched by magnetic selection. Cells were analyzed by 10X single cell RNA (scRNA) sequencing as described in figure 3a. The data shows differential gene expression with a q value of 0.05 cut off between *Tet2*-KO and WT HSCs as determined using DESeq2.

File Name: Supplementary Data 3
Description: **Enrichr and STRING ontology analysis in Tet2-KO relative to WT HSCs.** WT and *Tet2*-KO mice were treated for 5 weeks with and without IL1β (n = 4 mice/group). BM cells were harvested and lineage cells were depleted by magnetic selection. Cells were analyzed by 10X single cell RNA (scRNA) sequencing as described in figure 3a. Enrichr (KEGG 2021 Human), STRING KEGG Pathways (mmu04010), or STRING biological process gene ontology (GO:0034097) identified pathways from genes upregulated in *Tet2*-KO relative to WT HSCs treated with or without IL1β, or in both conditions. q values were determined through Enrichr and STRING.

File Name: Supplementary Data 4
Description: **Gene set enrichment analysis results for C2 molecular signature database in Tet2-KO relative to WT HSCs.** WT and *Tet2*-KO mice were treated for 5 weeks with and without IL1β (n = 4 mice/group). BM cells were harvested and lineage cells were depleted by magnetic selection. Cells were analyzed by 10X single cell RNA (scRNA) sequencing as described in figure 3a. Gene set enrichment analysis (C2) of differential expressed genes between HSCs in *Tet2*-KO and WT with and without IL1β administration. q values were determined by GSEA v4.2.1 using an empirical phenotype-based permutation test procedure.

File Name: Supplementary Data 5
Description: **List of differentially methylated regions between Tet2-KO and WT LSK, CMP and GMP cells.** WT and *Tet2*-KO mice were treated for 5 weeks with and without IL1β (n = 4 mice/group). BM cells were harvested and LSK, CMP and GMP cells were flow sorted and bisulfite sequenced as described in Supplementary Figure 7a. Data shows differentially methylated regions in *Tet2*-KO relative to WT LSK, CMP, and GMP cells in *Tet2*-KO relative to WT with and without IL1β administration and in the vehicle, relative IL1β treated mice. >10% methylation difference, $q < 0.05$, statistical significance determined by SeqMonk.

File Name: Supplementary Data 6
Description: **List of GEO accession numbers for ChIP-seq factors.** GEO accession numbers for ChIP-seq databases whose called peaks were acquired from cistrome.

File Name: Supplementary Data 7
Description: **HOMER motif analysis of differentially methylated regions between Tet2-KO and WT LSK cells.** Differentially methylated regions for fluorescently activated cell sorted LSK, CMP, and GMP cells described in Supplementary Data 4 underwent HOMER motif analysis for *Tet2*-KO relative to WT mice treated with vehicle or IL1β. Statistical significance of motif enrichment was determined by hypergeometric distribution using the HOMER analysis tool.
